# A Case of Phlegmonous Gastritis in a Patient with Psoriatic Arthritis on Infliximab

**DOI:** 10.1155/2018/3624627

**Published:** 2018-08-15

**Authors:** Laurence De Davide, Annie Beaudoin

**Affiliations:** Department of Gastroenterology, Sherbrooke University Hospital Center, Sherbrooke, QC, Canada

## Abstract

Phlegmonous gastritis is a pyogenic infection affecting the submucosa of the gastric wall. Although rarely diagnosed, it remains a disease with high mortality. We thereby describe the case of a 42-year-old male patient known for psoriatic arthritis on Infliximab who was diagnosed with phlegmonous gastritis secondary to immunosuppressive therapy. The patient had a favourable outcome with a conservative treatment consisting of a 14-day course of broad antibiotherapy.

## 1. Introduction

Phlegmonous gastritis remains a rare but serious entity although its prognosis has greatly improved with the advent of antibiotics. Described for the first time in 1862 by Cruveilhier [1], it is caused by a bacterial infection of the gastric submucosa [2]. Primary and secondary forms have been defined [3–5]. Streptococcus is implicated in approximately 70% of cases [6], but many cases are believed to be polymicrobial [7].

Its clinical presentation is nonspecific, including fever, epigastric pain, and nausea, making the diagnosis laborious. Purulent emesis has been known to be pathognomonic but has not been described in the recent series of cases on the subject [1, 7]. A palpable epigastric mass can be found [8, 9].

On CT scan, the gastric wall will be thickened due to neutrophilic and plasmatic infiltration, with a low intramural density [3]. Endoscopic ultrasonography is believed to be more precise and could help distinguish phlegmonous gastritis from other submucosal lesions [7, 10]. The mucosa will be thickened and erythematous on endoscopy, with loss of the gastric folds [1]. The disease can affect the whole stomach or can be localized, most commonly at the antrum [11].

## 2. Description of the Case

We report the case of a 42-year-old patient, known for psoriatic arthritis on Infliximab since one year. The patient had tried other immunosuppressive therapies, including methotrexate, which had been stopped because of a Child A cirrhosis. The patient's other medication included ezomeprazole, duloxetine, bupropion, olmesartan, tamsulosin, oxybutynin, zolpidem, and codeine if needed. The patient had not been taking nonsteroidal anti-inflammatory agents or corticotherapy recently and did not drink alcohol.

The patient presented himself at the emergency for epigastric pain, fever up to 40.2°C, nausea without vomiting, and hemodynamic instability necessitating temporary use of vasopressors. He had no known infectious contact, no history of recent travel, and no change in his diet. Upon arrival, the bloodworks demonstrated leukocytosis (14.4 x 10^9^/L) with neutrophilic predominance (12.1 x 10^9^/L) and thrombopenia (platelets 73 x 10^9^/L). Hemoglobin, creatinine, and liver function tests were normal.

**Figure 1:**
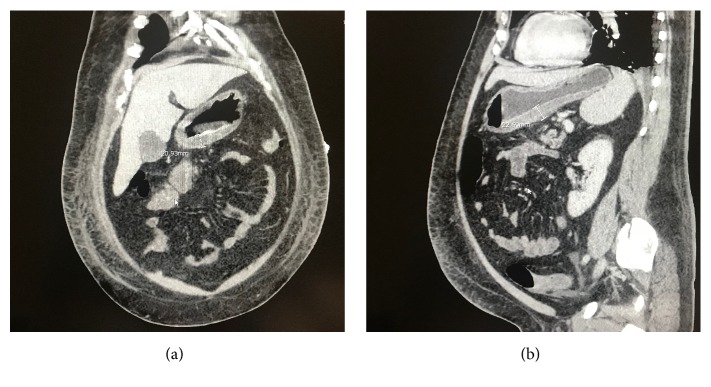
Thickening of the gastric submucosa on coronal (a) and sagittal (b) views.

A first CT scan showed thickening of the gastric antrum up to 2.2cm, with an inflammatory submucosa with liquid density (Figure 1). A splenomegaly was also noted. A diffuse gastric thickening was visualised on endoscopy, more prominent at the antrum. The patient was then referred to our tertiary center for further evaluation. He was placed on broad-spectrum intravenous antibiotics, with coverage for* S pyogenes* considering the high suspicion of phlegmonous gastritis based on the paraclinical findings. Ganciclovir was added to a regimen of imipenem, vancomycin, and clindamycin, for empirical coverage of CMV. The patient was placed on intravenous proton pump inhibitor.

Although the patient's condition improved, the control endoscopy five days later demonstrated once again the thickened, oedematous, and erythematous gastric mucosa, especially at the antrum, without ulcers. An extensive infectious workup did not reveal any pathogen and the pathology showed a nonspecific gastritis, with absence of* H pylori*, CMV, or neoplasia. Histology revealed a light neutrophilic inflammation with few eosinophils. Empirical antibiotic coverage was reduced to imipenem for a total of 14 days.

The patient recovered completely thereafter. Infliximab was definitely ceased, being replaced by sulfasalazine, under the recommendation of the rheumatology team.

## 3. Discussion

The main risk factor associated with phlegmonous gastritis is immunosuppression, in up to 50% of cases, including HIV, corticotherapy, malignancy, alcohol consumption, chronic illnesses, and immunosuppressive medications [1, 12]. In up to 41% of the cases, however, no risk factor can be identified [7]. Proton pump inhibitors and anti-H2 could also contribute to bacterial proliferation by increasing gastric pH [3, 13].

The fact that our patient was on Infliximab for psoriatic arthritis certainly contributed to the development of the phlegmonous gastritis. It is therefore crucial to question the upcoming management of his arthritis. There is only one other case of patient under Infliximab diagnosed with diffused phlegmonous gastritis reported in the literature that we are aware of [14]. This patient had ankylosing spondylitis and had been on Infliximab for six months. The paraclinical findings were similar to our patient except for the extent of the disease that included the whole stomach and for the fact that multiple ulcers were found at gastroscopy. The authors reported a favourable outcome under large spectrum antibiotic therapy alone for a total of three weeks. However, it remains unclear if the immunosuppressive treatment must be modified, relapses not being described in the literature. To our knowledge, no other case of phlegmonous gastritis was reported in a patient with psoriatic arthritis. A case of a patient with rheumatoid arthritis under a nonsteroidal anti-inflammatory agent was however described, with diagnosis made only at autopsy, the patient not wanting any investigations [13].

Since the first review of cases published in 1919, mortality rates have been decreasing, from 92% to 27-42% [1, 7], thanks to the evolution of antibiotic therapy. According to a recent review of 45 cases, no difference on mortality has been observed between antibiotic and surgical treatment [1]. Duration of antibiotic therapy remains however variable according to the case reports, extending from 7 to 21 days [3, 14–16]. Our patient had a favourable outcome with 14 days of a broad-coverage antibiotic.

## 4. Conclusion

Phlegmonous gastritis is a rare disease with high mortality despite optimal medical treatment, being frequently diagnosed too late. An early broad-spectrum antibiotic therapy seems to be the key to avoid surgery, which remains inevitable in some cases. Many questions remain to be answered to ensure optimal management of phlegmonous gastritis, but our case demonstrates that clues found on endoscopy and imaging can orient towards a quick diagnosis and large spectrum antibiotics can eradicate infection.

## Data Availability

No new data were created during this study.
